# Acute Uterine Torsion Masquerading as Fibroid Degeneration in a High-Risk Pregnancy: A Case of Diagnostic Surprise

**DOI:** 10.7759/cureus.87156

**Published:** 2025-07-02

**Authors:** Ajanta Samanta, Riya Bera, Medha Barua, Durlabh Debbarma, Arindam Pande

**Affiliations:** 1 Obstetrics and Gynecology, R. G. Kar Medical College and Hospital, Kolkata, IND; 2 Cardiology, Medica SuperSpecialty Hospital, Kolkata, IND

**Keywords:** arabin pessary, myomectomy in pregnancy, preterm labor and obstetric care, uterine fibroid in pregnancy, uterine torsion

## Abstract

Uterine torsion during pregnancy is a rare and life-threatening condition that is frequently misdiagnosed due to nonspecific symptoms and imaging limitations. This case report describes a 32-year-old third gravida (G3, P0+2) with recurrent pregnancy loss, having a 16.9 cm uterine fibroid and hypertrophic cardiomyopathy (managed with bisoprolol), who presented at 24 weeks with severe abdominal pain. Although her vitals were stable, a markedly elevated C-reactive protein (CRP) (245 mg/dL) raised concern for acute pathology. Initial ultrasound incorrectly localized the fibroid to the left, but exploratory laparotomy revealed a 180-degree uterine torsion with contralateral fibroid position, revising the diagnosis from fibroid degeneration to this rare emergency, leading to detorsion and myomectomy. At 30 weeks' gestation, cervical insufficiency (a short cervix measuring 0.5 cm with funneling) was successfully managed with an Arabin pessary and weekly 500 mg injections of hydroxyprogesterone, prolonging the pregnancy to 34 weeks and resulting in an outlet forceps delivery of a healthy 1.9 kg infant. This case highlights the importance of surgical exploration when clinical suspicion contradicts imaging findings, the feasibility of pregnancy-preserving surgery for uterine torsion, and the effectiveness of combined mechanical-hormonal therapy for cervical insufficiency following complex uterine interventions. Multidisciplinary care was critical to manage overlapping high-risk factors, including fibroids, cardiac disease, and preterm cervical changes, ultimately leading to a favorable outcome.

## Introduction

Uterine torsion, defined as a rotation of the uterus greater than 45° around its longitudinal axis, is a rare but potentially life-threatening obstetric emergency associated with significant fetal risks if diagnosis is delayed [1]. Diagnostic challenges are compounded by the nonspecific clinical presentation and the limited sensitivity of ultrasound for detecting torsion, often leading to misdiagnosis as fibroid degeneration. This delay in surgical intervention can result in perinatal mortality rates as high as 12% [2,3]. These difficulties are particularly pronounced in patients with large fibroids, as the mechanical distortion of uterine anatomy increases the risk of torsion and can lead to catastrophic obstetric emergencies, such as massive placental abruption [4]. Our case exemplifies these challenges in a third gravida patient with a 16.9 cm fibroid and bisoprolol-treated hypertrophic cardiomyopathy, where classic signs were masked and only an isolated elevation in C-reactive protein (CRP), recently proposed as a diagnostic marker, prompted surgical intervention [5].

## Case presentation

**Figure 1:**
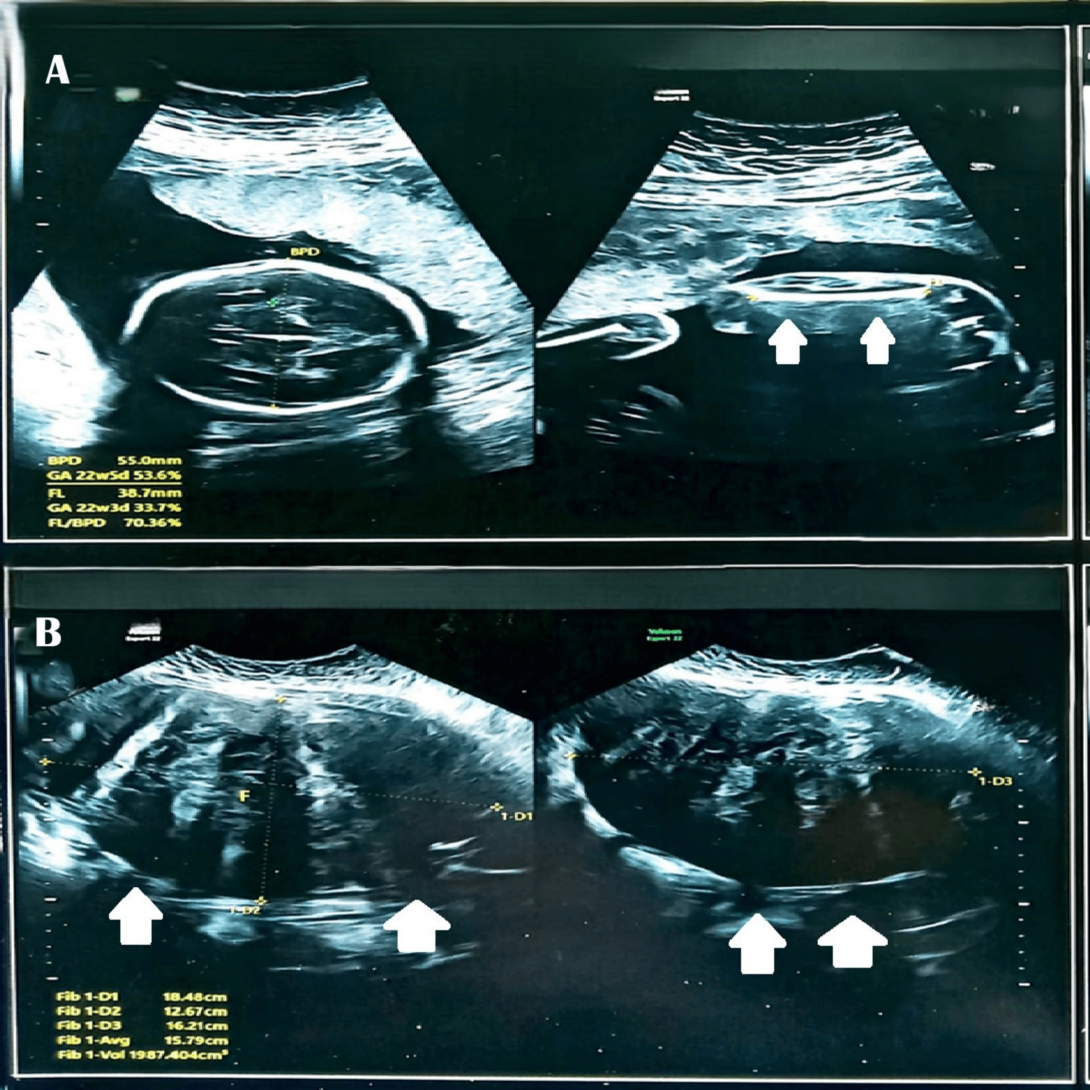
Ultrasonographic findings at 22 weeks of gestation complicated by a giant uterine fibroid. (A) Composite image showing fetal biparietal diameter (BPD) and femur length (FL) measurements, marked by double white arrows. (B) Transabdominal ultrasound demonstrating a large intramural fibroid (FIGO type 5) measuring 18.48 × 12.67 × 16.21 cm with heterogeneous echotexture, also marked by double white arrows. The images highlight the technical challenges of sonographic fetal monitoring in the presence of a giant fibroid. Notably, the fibroid’s location and size did not impair standard biometric assessments, underscoring the importance of meticulous imaging planes to distinguish fetal structures from fibroid tissue.

A 32-year-old third gravida (P0+2) with two prior spontaneous miscarriages (at 3 months and 1 month gestation) presented with a high-risk pregnancy complicated by a large uterine fibroid (16.9 × 9.8 × 14.5 cm) detected at nine weeks' gestation. Her medical history included hypertrophic cardiomyopathy (managed with bisoprolol since 2017) and hypothyroidism (on thyroxine 175 mcg since 2017). Due to the fibroid's size, nuchal translucency (NT) scan could not be performed, but non-invasive prenatal testing (NIPT) showed no chromosomal abnormalities. The patient declined myomectomy due to fetal risks, opting for conservative management. At 22 weeks of gestation, ultrasonography revealed a large intramural uterine fibroid (International Federation of Gynecology and Obstetrics (FIGO) type 5) measuring 18.48 × 12.67 × 16.21 cm with heterogeneous echotexture (Figure 1).

At 24 weeks of gestation, she presented with five days of severe abdominal pain. Despite stable vital signs (likely masked by bisoprolol), examination revealed a tender abdominal mass. Laboratory results showed elevated CRP (245 mg/L), suggesting inflammation. Ultrasound failed to detect torsion, leading to the initial misdiagnosis of fibroid degeneration. Preoperative and early pregnancy ultrasounds had consistently localized the fibroid to the left uterine fundus, with severe left-sided tenderness supporting the degeneration diagnosis.

During pregnancy, fibroid degeneration is typically managed conservatively. However, severe unrelieved abdominal pain and tenderness with a rapidly rising CRP prompted surgical intervention. A multi-disciplinary team, including a cardiologist, a critical care specialist, and an anesthesiologist, was involved during the surgery and delivery.

**Figure 2:**
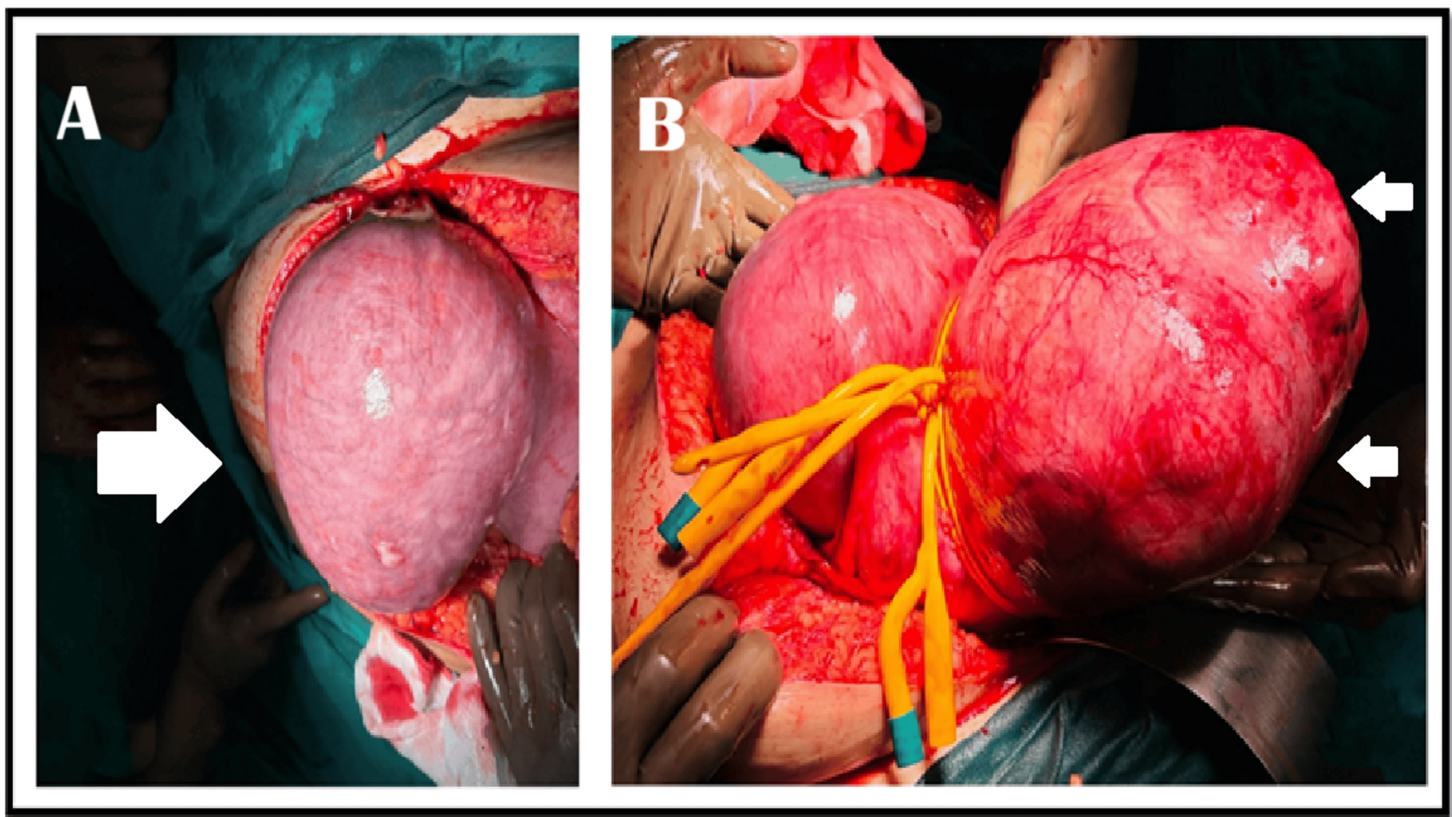
Composite image (A) demonstrates the gestational sac (large white arrow), which was initially misdiagnosed as a fibroid with degeneration, and (B) shows the fundal fibroid (double small white arrows) after extension of the incision and application of a Foley catheter as a tourniquet to minimize blood loss, as vasopressin is contraindicated in the presence of a live fetus.

**Figure 3:**
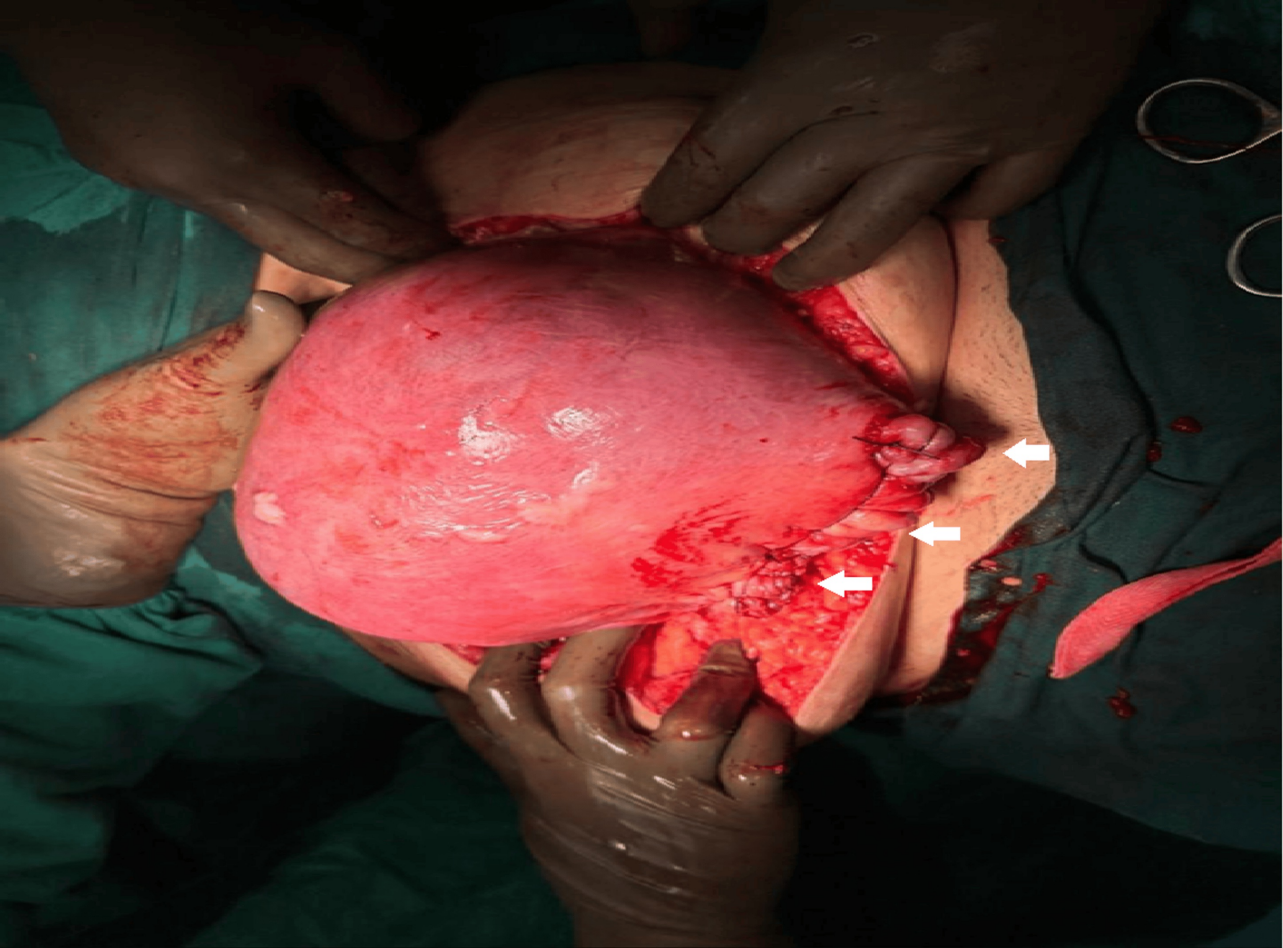
The post-myomectomy image shows successful preservation of the pregnancy, with triple small white arrows indicating the final repair site of the myomectomy scar.

Exploratory laparotomy through an infraumbilical vertical incision initially revealed a soft, discolored left-sided mass presumed to be a degenerated fibroid. However, upon extending the incision beyond the umbilicus, we discovered a 180° uterine torsion, with the fibroid located on the right side. The initial finding represented the inflamed gestational sac. Detorsion was performed, followed by successful myomectomy using dual Foley catheters as tourniquets (vasopressin being contraindicated), with meticulous preservation of the gestational sac (Figures 2-3).

**Figure 4:**
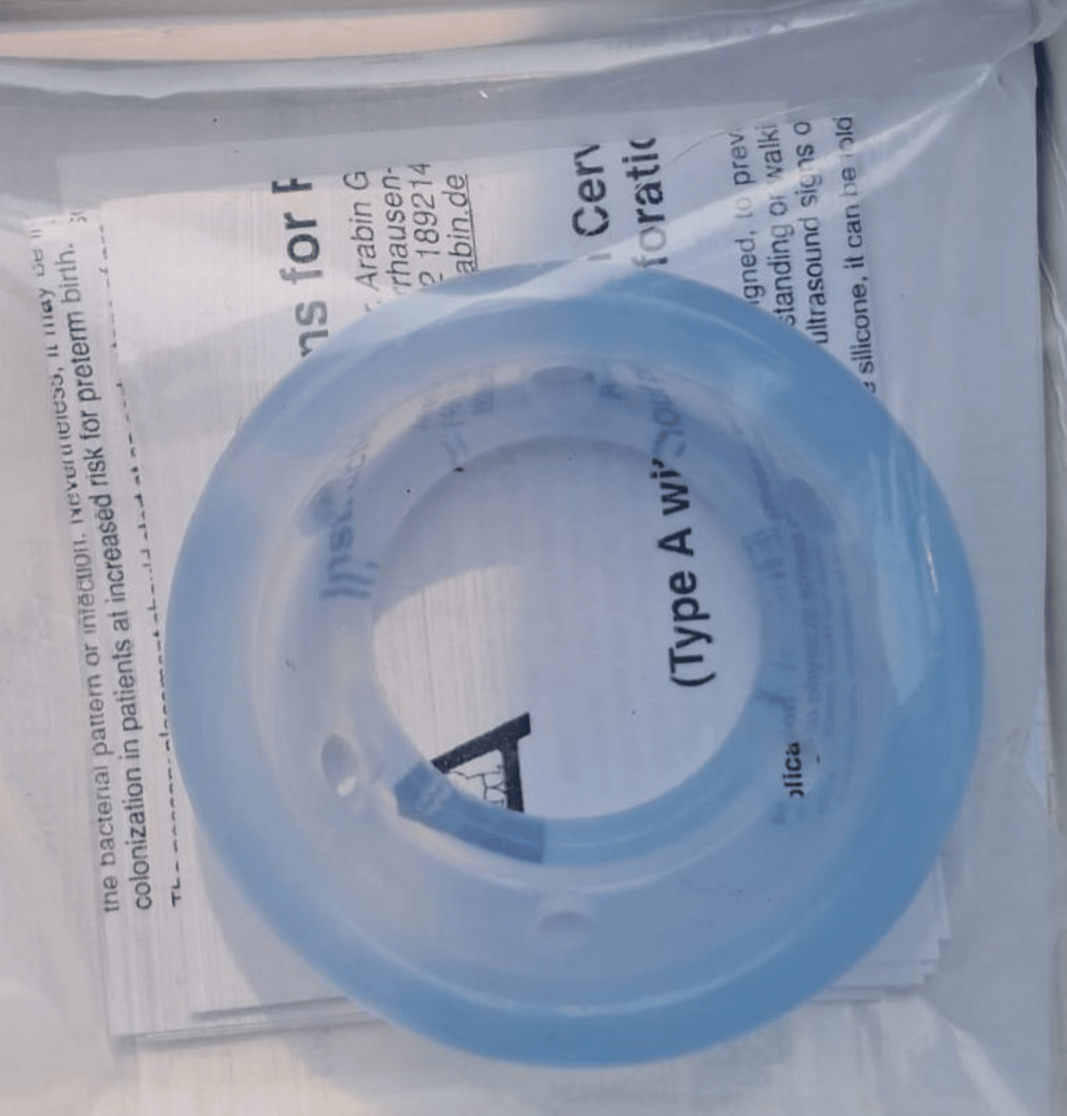
Image of the Arabin pessary (Dr. Arabin, Witten, Germany) inserted per vaginam for the prevention of preterm labor in the context of a short cervix at 30 weeks' gestation. The image was captured by the authors and is published under a Creative Commons license.

At 30 weeks, cervical incompetence developed (cervical length 0.5 cm with funneling). An Arabin pessary (Dr. Arabin, Witten, Germany) was inserted, and weekly 500 mg hydroxyprogesterone injections were administered, as tocolytics were contraindicated due to the patient's cardiac status. These interventions prolonged the pregnancy to 34 weeks, at which point a prophylactic outlet forceps delivery was performed, resulting in the birth of a healthy 1.9 kg infant. The neonate has thrived, showing good progress at one month postnatally. Informed consent was obtained for this case presentation (Figure 4).

## Discussion

Uterine torsion with coexisting large fibroids presents significant diagnostic and management challenges. This case demonstrates three critical insights: the utility of CRP as a surgical indicator when beta-blockers mask classical signs, the limitations of imaging in distorted anatomy, and the effectiveness of multidisciplinary strategies in high-risk pregnancies.

Our patient's bisoprolol-masked tachycardia correlates with previous reports of medication-concealed torsion signs [6]. The markedly elevated CRP (245 mg/L) served as a crucial indicator for surgical intervention, supported by findings of a previous study where raised CRP levels guided prompt surgical management [5]. However, unlike typical torsion cases where imaging provides definitive diagnosis, our patient's FIGO type 5 large fibroid required intraoperative confirmation, reinforcing the caution mentioned in the earlier study regarding imaging interpretation in complex fibroid cases [7]. Although radiological imaging provides valuable guidance, especially when performed intraoperatively, this case underscores that clinical judgment must remain paramount, with imaging serving as an adjunct rather than a replacement for surgical assessment.

The patient had presented with a partially intramural fibroid (FIGO type 5) exhibiting a broad-based stalk. Thus, intraoperative use of a Foley catheter as a tourniquet during dissection of the upper portion until the fibroid base was approached effectively minimized blood loss, as vasopressin administration was contraindicated due to the viable intrauterine pregnancy supported by a previous study evaluating different methods of myomectomy [8]. Although at 30 weeks of gestation, emergency cervical cerclage was clinically indicated; however, given the patient’s recent uterine surgery and her residence being far from the hospital (posing a substantial risk of scar rupture), conservative management was pursued. The successful extension of gestation from 30 to 34 weeks was achieved through a tailored approach combining an Arabin pessary with progesterone supplementation (Injection Hydroxyprogesterone 500 mg weekly), as conventional tocolytics were contraindicated due to the patient’s hypertrophic cardiomyopathy [9]. This strategy effectively balanced prematurity risk reduction with cardiovascular safety. The decision for outlet forceps delivery addressed two concurrent risks: uterine rupture from the recent myomectomy (a 10-week-old scar) during maternal expulsive efforts, and hemodynamic instability from prolonged labor in the setting of cardiomyopathy. The forceps blades served a dual purpose of providing cranial protection for the preterm fetus while minimizing second-stage duration to reduce cardiac strain [10]. This outcome underscores how multidisciplinary collaboration (obstetric, surgical, and cardiology) can optimize results in complex cases where standard protocols require modification due to maternal comorbidities.

## Conclusions

This case report provides a comprehensive paradigm for managing uterine torsion in high-risk pregnancies, demonstrating how elevated CRP (245 mg/L) served as a critical surgical indicator when bisoprolol masked typical signs, while highlighting imaging's limitations in distorted anatomy. Though the conclusions are drawn from an isolated case, more data are needed to validate critically raised CRP as an indicator of surgical intervention or beta-blocker induced masking of obstetrical decompensations. The index case also presents novel technical solutions, including Foley catheter tourniquets for hemorrhage control during pregnancy-preserving myomectomy (when vasopressin was contraindicated) and a successful combined approach using Arabin pessary with progesterone supplementation to prolong gestation despite cervical insufficiency. The case makes three key contributions: first, establishing a pathophysiology-based rationale for prophylactic outlet forceps in addressing concurrent risks of uterine rupture, cardiac strain, and preterm delivery; second, demonstrating how traditional obstetric techniques retain vital relevance when applied with precise indication; and third, providing a multidisciplinary management model that achieved delivery of 1.9 kg neonate, despite 180° torsion and multiple comorbidities, ultimately emphasizing that optimal outcomes require clinical judgment to supersede conflicting radiological findings while systematically integrating surgical, mechanical, and pharmacological interventions tailored to complex presentations.

## References

[1] Bui TT, Le TV, Nguyen OT, Tran HT, Nguyen TD (2024). Uterine torsion in pregnancy: a case report. Int J Surg Case Rep.

[2] Liang R, Gandhi J, Rahmani B, Khan SA (2020). Uterine torsion: a review with critical considerations for the obstetrician and gynecologist. Transl Res Anat.

[3] Moores KL, Wood MG, Foon RP (2014). A rare obstetric emergency: acute uterine torsion in a 32-week pregnancy. BMJ Case Rep.

[4] Sachan R, Patel ML, Sachan P, Arora A (2014). Complete axial torsion of pregnant uterus with leiomyoma. BMJ Case Rep.

[5] Kim HJ, Lee J, Lee HJ (2022). Differential diagnosis of uterine leiomyoma torsion mimicking ovarian torsion in a second trimester of pregnancy: a case report. Int J Womens Health.

[6] Martinez A, Lakkimsetti M, Maharjan S (2023). Beta-blockers and their current role in maternal and neonatal health: a narrative review of the literature. Cureus.

[7] Le D, Dey CB, Byun K (2020). Imaging findings of a torsed pedunculated uterine leiomyoma: a case report. Radiol Case Rep.

[8] Incognito GG, Gulino FA, Cianci S (2024). Minimizing blood loss in laparotomic myomectomy through the tourniquet use: insights from our clinical experience and literature review. Surgeries.

[9] Sieroszewski P, Jasiński A, Perenc M, Banach R, Oszukowski P (2009). The Arabin pessary for the treatment of threatened mid-trimester miscarriage or premature labour and miscarriage: a case series. J Matern Fetal Neonatal Med.

[10] Easter SR, Rouse CE, Duarte V (2020). Planned vaginal delivery and cardiovascular morbidity in pregnant women with heart disease. Am J Obstet Gynecol.

